# Secondary Metabolites of *Saussurea costus* Leaf Extract Induce Apoptosis in Breast, Liver, and Colon Cancer Cells by Caspase-3-Dependent Intrinsic Pathway

**DOI:** 10.1155/2020/1608942

**Published:** 2020-07-12

**Authors:** Ali A. Shati, Mohammed A. Alkahtani, Mohamed Y. Alfaifi, Serag Eldin I. Elbehairi, Fahmy G. Elsaid, Rajagopalan Prasanna, Mushtaq A. Mir

**Affiliations:** ^1^Biology Department, Faculty of Science, King Khalid University, Abha 9004, Saudi Arabia; ^2^Cell Culture Lab, Egyptian Organization for Biological Products and Vaccines (VACSERA Holding Company), 51 Wezaret El-Zeraa St., Agouza, Giza 12311, Egypt; ^3^Zoology Department, Faculty of Science, Mansoura University, Mansoura, Egypt; ^4^Department of Clinical Laboratory Sciences, College of Applied Medical Sciences, King Khalid University, P. O. Box 3665, Abha 61421, Saudi Arabia

## Abstract

**Background:**

Apoptosis, a major form of programmed cell death, plays a vital role in regulating tissue development and maintenance of homeostasis in eukaryotes. Apoptosis can occur via a death receptor-dependent extrinsic or a mitochondrial-dependent intrinsic pathway and can be induced by various chemotherapeutic agents. In this study, the anticancer activity of *Saussurea costus* and its mode of intervention in human cancer cells of breast, colon, and liver were investigated.

**Results:**

In this study, the bioactives of *S. costus* leaves were extensively extracted in five solvents of different polarity. The cytotoxicity and anticancer effect of the extracted secondary metabolites were investigated against breast (MCF-7), liver (HepG2), and colon (HCT116) cancer cell lines using a Sulphorhodamine B (SRB) assay. Secondary metabolites extracted using hexane, methanol, ethyl acetate, and chloroform had the highest cytotoxicity and thus the greatest anticancer effect on all the cancer cell lines tested (IC_50_; ranging from 0.25 to 2.5 *μ*g/ml), while butanol was comparatively less active (IC_50_; ranging from 23.2 to 25.5 *μ*g/ml). Further investigation using DNA flow cytometry and fluorescent microscopy revealed that the extract arrested the cells in the G_1_ phase of cell cycle and induced apoptosis. Furthermore, the elevated expression level of proapoptotic proteins and decreased expression level of antiapoptotic proteins confirmed that the intrinsic (mitochondrial) pathway was involved in mediating the apoptosis of cancer cells upon treatment with *S. costus* extract. These results altogether suggest that *S. costus* could be a potential anticancer agent.

**Conclusion:**

These results suggest that the *S. costus* extract is the potential source of the secondary metabolites that could be used as anticancer agent to treat diverse cancers of breast, colon, and liver.

## 1. Background

Cancer, abnormal growth of cells, also called malignancy, is the second leading cause of death globally and is responsible for an estimated 9.6 million deaths in 2018 [1]. The most common cancers are lung, breast, liver, colorectal, prostate, skin, and stomach.

Hepatocellular carcinoma (HCC), the most common primary malignancy of the liver, is a leading cause of death in people with cirrhosis [2]. Chronic liver disease and cirrhosis by viral hepatitis remain the important risk factors for the development of HCC [3]. Significant progress has been made in diagnosis and treatment using multidisciplinary approaches of surgical (resection, liver transplant), nonsurgical transarterial chemoembolization, transarterial radiation, percutaneous local ablation, microwave ablation [4], and systematic chemotherapy [5]. Breast cancer, the most common cancer among women and prevalent in underdeveloped countries, is one of the leading causes of morbidity and mortality for women worldwide [6]. Colorectal cancer is a multifactorial disease, of which several risk factors have been identified involving genetic and environmental factors, lifestyle, and gut microbiota. Though emerging chemotherapeutic agents inhibiting the cellular pathways involved in the cellular proliferation have revolutionized the treatment of various cancers, the universal drug resistance development demands the generation of new therapeutic agents [7].

A variety of cytotoxic drugs reported to combat cancer usually are incompetent not only because of their efficacy but also because of their undesirable side effects. There is a need for novel anticancer compounds without adverse side effects. Plants are known to have a long history in cancer treatment [8] and medical practices. The use of plants and their derived products for the treatment of cancer is rapidly growing [9]. In this study, various polar and nonpolar solvent extracts were prepared from the leaves of *S. costus* to possibly isolate almost all bioactives, and then, these diverse solvent extracts were tested individually against diverse cancer cell lines.

*Saussurea costus* (Costaceae family), habitant of the subalpine region of Jammu and Kashmir, Himachal Pradesh, and Uttarakhand, is used to treat various ailments, *viz.*, ulcer, headache, rheumatism, cough and cold, and throat infection, while in Korea it is frequently used in Korean traditional prescriptions for inflammatory diseases [10]. Its anticancer [11–13] activity, including other activities of anti-inflammatory [14, 15], antioxidant [16, 17], antifungal, and antibacterial [18], has gained the attention of the scientific community to explore the underlying mechanisms of aforesaid activities. Its extract inhibits the cytokine-induced neutrophil chemotactic factor (CINC) induction [19] and tumor necrosis factor- (TNF-) alpha production in LPS-stimulated RAW264.7 cells [20]. Costunolide, an active compound isolated from *S. lappa* roots, has anticancer activity by inhibiting the proliferation of HL-60 human leukemia cells by induction of apoptosis through ROS-mediated mitochondrial permeability transition and cytochrome C release [21]. In another study, costunolide showed anticancer activity against human lung squamous carcinoma (SK-MES 1) cells by inducing G_1_/S phase arrest and activating mitochondrial-mediated apoptotic pathways [22]. All these beneficial uses including anticancer effects of a taxonomically related *S. costus*, commonly known as kuth in Kashmiri, have not been investigated particularly in the cancer cell lines taken in this study.

Apoptosis, a major form of programmed cell death, plays a vital role in regulating tissue development and maintenance of the homeostasis in eukaryotes [23–25]. One of the hallmarks of cancer is the deregulation of apoptosis [25]. Therefore, an effective mode of intervention for chemopreventive and chemotherapeutic agents in many types of cancers could be the upsurge of apoptosis. Apoptosis can occur via a death receptor-dependent extrinsic or mitochondrial-dependent intrinsic pathway and can be induced by various chemotherapeutic agents [26].

In this study, we explored the possibility whether *S. costus* could function as a chemotherapeutic agent in human cancers. We tested *S. costus* extracts for their effects in inhibiting cell proliferation of the cancer cells of breast, liver, and colon and characterized its underlying mechanisms of action. The demonstration of anticancer activity of the *S. costus* extracts against diverse cancer cell lines in this study indicates that the plant extract/its bioactives can be used in the treatment of diverse cancers. However, isolation of active compounds and their anticancer activity needs to be tested *in vivo* using animal models.

## 2. Results

### 2.1. Effect of Extract on the Proliferation of Cancer Cell Lines

**Figure 1:**
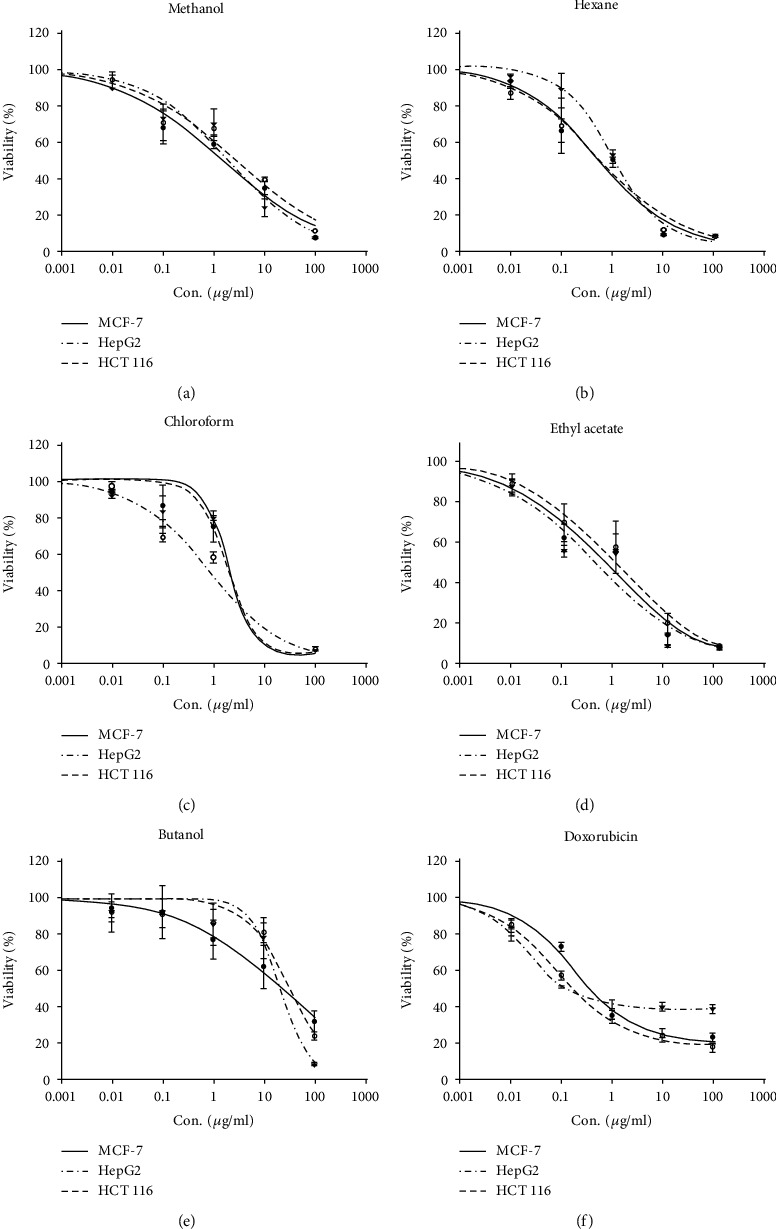
IC_50_ values of the *Saussurea costus* extracts against cancer cell lines. The graph plotted between the percentage of viable cells of the cell lines MCF-7, HepG2, and HCT116 treated against the various concentrations (*μ*g/ml) of extracts, *viz.*, (a) methanol, (b) hexane, (c) chloroform, (d) ethyl acetate, (e) butanol, and the drug (f) doxorubicin. The percentage of viable cells was determined by SRB assay after 72 hours of treatment with an extract or the drug doxorubicin.

In order to detect the effect of different solvent extracts of *S. costus* on the proliferation of different types of cancer cell lines, an SRB assay was performed. The viability (%) of the cancer cells was used as an indicator of the cell toxicity towards the treatment of cells with different concentrations of extract for 72 hrs. The results demonstrate that the viability of cells decreased in a dose-dependent manner (Figure 1), potentially killing all cells at 100 *μ*g/ml. A gradual increase in the dose of an extract resulted in the gradual increase in the growth inhibition of all three types of independently treated tumor cells. The cytotoxic activity of methanol, hexane, chloroform, and ethyl acetate was very strong (IC_50_; 0.5–2.5 *μ*g/ml), almost similar to that of a prominent anticancer drug, doxorubicin (IC_50_ < 1 *μ*g/ml). The methanolic extract exhibited comparatively less cytotoxic effect (IC_50_; 25 to 32.2 *μ*g/ml) against all the cell lines tested (Table 1). IC_50_ values of the extracts, except methanol, being within the closer range to that of doxorubicin prompted us to look for the underlying mechanism of their cytotoxicity.

### 2.2. *S. costus* Extract Induced Cell Cycle Arrest

**Figure 2:**
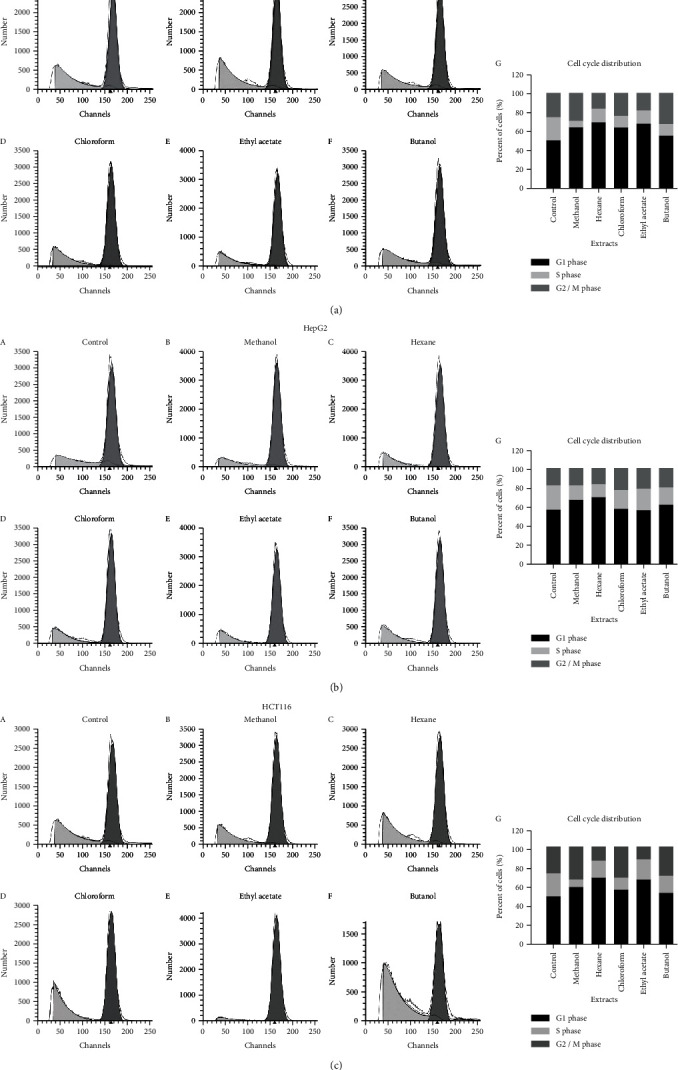
Cell cycle analysis of cancer cell lines after *Saussurea costus* extract treatment. The cell cycle distribution of cells of the cell lines (a) MCF-7, (b) HepG2, and (c) HCT116 analyzed by DNA flow cytometry after their treatment with a plant extract. Bar graphs show the percentage of cells of a cancer cell line in different phases of the cell cycle after its treatment with a plant extract.

Cell cycle arrest is one of the major causes of inhibition of cellular growth. To determine whether the inhibition of cellular growth was due to the arrest of the cell cycle at a specific phase of the cell cycle, the cell lines were individually treated with the extracts at their IC_50_ concentrations, and then, the cell cycle profile was determined by PI staining followed by DNA flow cytometry analysis. As shown in Table 2 and Figure 2, the significant population of all the tumor cell lines, except HepG2, was found in the G_1_ phase of the cell cycle after their treatment with either methanol or hexane or ethyl acetate, compared to the control where cells were treated with the DMSO only. Rather than increasing the HepG2 cellular population in the G_1_ phase, there was a significant increase in the population of cells in the G_2_/M phase upon their treatment with ethyl acetate compared to the control. Chloroform displayed varying effects on the cell lines tested. Compared to control, chloroform treatment significantly increased the MCF-7 and HepG2 cell population in G_1_ and G_2_/M phases, respectively, while as compared to control a significant population of HCT116 cells was observed in G_1_ and G_2_/M phases of the cell cycle upon their treatment with chloroform. Butanol, on the other hand, exhibited markedly increased cell population of all cell lines in both G_1_ and G_2_/M phases. The results demonstrate that all the extracts basically arrested all the types of cancer cells in the G_1_ and G_2_/M phases of the cell cycle, though the percentage of cells showed a variation.

### 2.3. *S. costus* Extracts Induced Apoptosis in Cancer Cells

**Figure 3:**
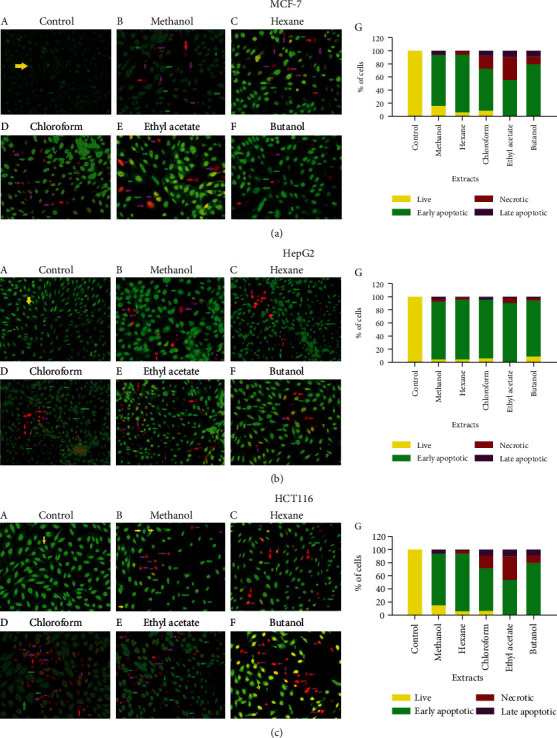
*Saussurea costus* extracts induce apoptosis. The representative fluorescent images of the cancer cell lines (a) MCF-7, (b) HepG2, and (c) HCT116 treated with a plant extract for 48 hours and stained with AO/EB. (G) Bar graphs show the percentage of cells of a cell line in different stages of apoptosis after its treatment with a plant extract. Yellow arrow: live cell; green arrow: early apoptotic; red arrow: necrotic; purple arrow: late apoptotic.

To further investigate the extract-induced inhibitory effect, cells treated with various extracts were analyzed under a fluorescent microscope for nuclear morphological changes (apoptosis or necrosis) after acridine orange/ethidium bromide staining. The major hallmarks of apoptotic cell death are DNA fragmentation and loss of membrane asymmetry. As shown in Figures 3(a)–3(c) (subpanels A–F), the extracts of *S. costus* induced morphological changes, DNA fragmentation, nuclear shrinking, etc., which are characteristics of various stages of apoptosis, *viz.*, early or late phase apoptosis. Analysis of the percentage of cells detected in different stages of apoptosis (subpanel G) suggested that the major cellular populations of all tumor cell lines tested against all types of extracts were in the early apoptotic stage. However, a marked percentage of HCT116 cells were in the necrotic stage of apoptosis when treated individually with chloroform, ethyl acetate, and butanol.

### 2.4. Apoptosis of Cancer Cells Occurs through the Executioner Caspase-3

**Figure 4:**
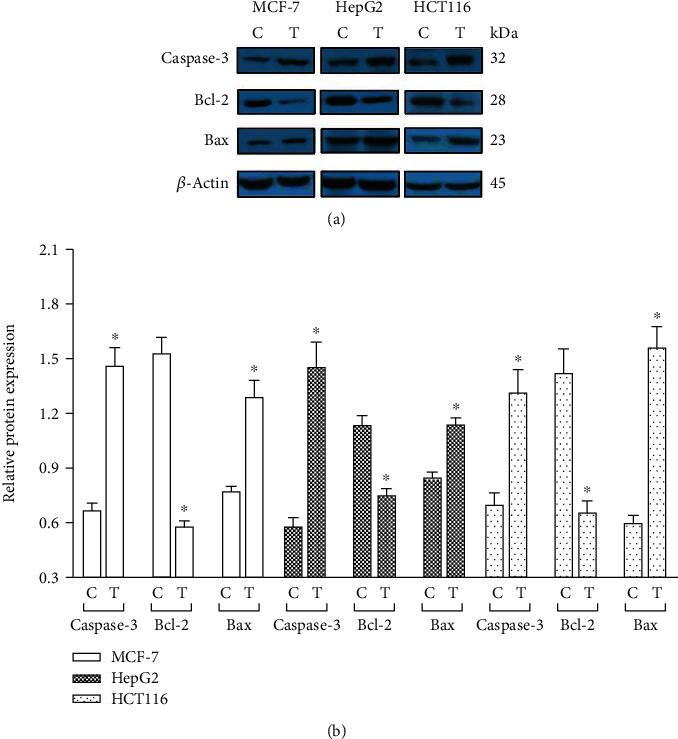
Expression profile of Bcl-2, Bax, and caspase-3. (a) Western blot analysis of Bcl-2, Bax, and caspase-3 proteins in the cell lysates of MCF-7, HepG2, and HCT116 obtained after 48 hrs of treatment with *S. costus* hexane extract. (b) Relative protein expression level of Bcl-2, Bax, and caspase-3 in control (C) and extract-treated (T) MCF-7, HepG2, and HCT116 cells. ∗*P* < 0.05.

The disturbance of apoptotic molecular signaling pathways is involved in carcinogenesis. In the programmed cell death pathway (apoptosis), the activation of the executioner caspase-3 and caspase-7 is regulated both by extrinsic (death ligand) and intrinsic (mitochondrial) pathways. The BCL2 family of proteins, including both antiapoptotic (Bcl-2) and proapoptotic (Bax) regulators, is the hallmark of apoptosis regulation. We, therefore, addressed the potential mechanism by which *S. costus* extract was causing decreased cell viability in all the cancer cell lines tested. For this purpose, we tested the hexane extract for having the least IC_50_ for all the four cancer cell lines. The level of Bcl-2 (antiapoptotic) and Bax (proapoptotic) expression in cancer cells was determined following treatment with the hexane extract of *S. costus*. Treatment of *S. costus* extract decreased the levels of Bcl-2 in all cancer cells, while the level of Bax expression is highly induced after 48 hours of treatment (Figure 4). It has been known that the caspase family activation represents one of the earliest known steps in the programed cell death process. Next, we explored the induction of caspase-3 upon the treatment of cancer cells with *S. costus* extract. Cancer cells exposed to *S. costus* extract exhibited the robust activation of caspase-3 (Figure 3).

## 3. Discussion

Although significant progress has been made in medical technology, there is no cure for almost any cancer around the globe. Natural products, which are used for the treatment of various ailments, are obtained from medicinal plants. These products, extracted in their crude form or as purified compounds, have been used as bases for discovering new drugs. Because of the chemical diversity of natural products, the interest and demand to identify the actual effective compound have grown globally [27]. Plant extracts and chemically synthesized molecules have been investigated in vitro for antibacterial as well as anticancer activity [28, 29]. Herbal medicine treatment provides some advantages over the use of single purified chemicals [30]. This could be due to the presence of mixtures of different therapeutic or preventive compounds or components, which could be more active in treating diseases than single products on their own [31]. In this study, the cytotoxicity properties of various *S. costus* extracts have been investigated for their biological efficacy against cancer cells. The dried plant leaves were used for crude extract preparation using different solvents. We used several solvents (methanol, hexane, ethyl acetate, butanol, and chloroform) to extract all compounds of varying polarity. The four solvent extracts methanol, hexane, chloroform, and ethyl acetate showed strong cytotoxic activity against all the three cancerous cell lines tested (IC_50_ < 2.6 *μ*g/ml). However, butanol was comparatively less effective (IC_50_; 25–32 *μ*g/ml). Cell cycle analysis showed that the extracts basically arrested all types of cancer cells in the G_1_ or G_2_/M phase of the cell cycle, though the percentage of cells showed a variation with respect to a particular cell line (Table 2, Figure 2). Studies show that anticancer molecules arrest cells in the growth phase of the cell cycle and subsequently induce cell death by apoptosis [32]. It has been proven that sequence of events occurs as the damaged cells progress through cell cycle arrest into G_1_ or G_2_/M phases which then pass through aberrant mitosis to subsequently undergo apoptosis [33]. This type of apoptosis usually occurs as a later event in cell death resulting from several distinct pathways. Certain anticancer agents are shown to cause DNA damage, which results in stagnation of cells in the G_1_ or G_2_/M phase before inducing apoptosis [34]. The present results showed cell cycle arrest by the extracts in either of the aforementioned phases of the cell cycle, thereby suggesting the association of apoptosis-inducing property of the extracts in the tested cancer cells. The control cells displayed normal and round nuclei with faint fluorescence, while cells treated with extracts showed condensed/fragmented and highly fluorescent nuclei (Figure 3). The observed physiological changes in the nuclei of extract-treated cancer cells suggested features of apoptosis. A crude extract exhibiting the IC_50_ value of less than 50 *μ*g/ml is said to have antitumor properties [35], suggesting that the *S. costus* extracts have strong anticancer effects. Further, we explored the pathway that is involved in mediating the apoptosis of cancer cells.

The BCL-2 protein family is a large family of proteins, which regulates apoptosis by modulating the mitochondrial pathway. It includes antiapoptotic and proapoptotic proteins such as Bcl-2 and Bax [36]. To explore the molecular mechanisms responsible for *S. costus* extract-induced apoptosis in cancer cells, *viz*., breast, liver, and colon, the expression of Bax and Bcl-2 protein in all the three types of cancer cells was examined. We examined the expression of Bax, Bcl-2, and Caspase-3 after treating the cancer cells with hexane extract of *S. costus* because the hexane extract of *S. costus* showed the best IC_50_ for all the cell lines tested. The results demonstrated that the expression of Bax increased and that of Bcl-2 decreased by 48 hours of treatment with the extract (Figure 4), indicating that the *S. costus* extract plays a critical role in the apoptosis of cancer cells. It is well known that the increased expression of Bax increases the mitochondrial membrane permeability, which leads to the release of cytochrome C and other proapoptotic molecules, thus activating downstream caspases and ultimately Caspase-3 [37]. In our study, increased expression of Caspase-3 was observed in the extract-treated cells (Figure 4). These results are in agreement with the previous study, wherein it was demonstrated that *S. costus* induces growth inhibition and apoptosis of human gastric cancer cells by down- and upregulation of growth-regulating apoptotic and tumor suppressor genes, respectively [38]. In another similar study, costunolide, a bioactive of *S. lappa*, induced micronuclei formation, chromosomal aberrations, and mitochondrial-mediated apoptosis of Chinese hamster ovary cells [39].

These results demonstrate that the mitochondrial-mediated caspase activation pathway is involved in extract-mediated apoptosis of human cancer cell lines of breast, liver, and colon. However, the role of the extrinsic pathway or ROS generation cannot be ruled out. More investigation is required to determine the role of other regulators/signals involved in the cytotoxic and apoptotic effect of the *S. costus* extracts.

## 4. Conclusion

In conclusion, the extract induced apoptosis in human cancer cells accompanied by a marked accumulation of cells in the G_1_/G_2_/M phase of the cell cycle to favor apoptosis. Apoptotic induction of the extracts was due to the upregulation of Bax and downregulation of Bcl-2 and caspase-3. These findings suggest that *S. costus* extract contains bioactives that might act potential therapeutic agents for the treatment of breast, liver, and colon cancers. However, further evaluations, active compound isolations, and *in vitro* and *in vivo* evaluations are recommended for future research on these active ingredients.

## 5. Materials and Methods

### 5.1. Extraction and Crude Extract Preparation

The dry leaves of *S. costus* were obtained from a herbal store (Khamis Mushayt, Saudi Arabia). For the fresh crude extract preparation, 100 g of fresh leaves was washed with distilled water and ground by a grinder with 500 ml of 80% aqueous ethanol. The leaves were then immersed in one liter of 80% aqueous ethanol, left for seven days at room temperature (18–24°C), and occasionally stirred. The ethanol extract was filtered using filter paper and concentrated to dryness under reduced pressure using a rotary evaporator at 37°C (Ika, Deutschland, Germany). The concentrated crude extract weight was 10 g, and the extraction yield was 10%. The crude extract was then reconstituted in 400 ml of distilled water and extracted with different solvents according to the polarity, *viz.*, methanol, hexane, chloroform, ethyl acetate, and butanol (hexane, ethyl acetate, chloroform, and n-butanol are nonpolar, semipolar, and polar, respectively), using liquid-liquid extraction method. After that, the solvent phase was separated and reevaporated using a rotary evaporator. The solvent was then left at room temperature (20–26°C) for complete evaporation for 3 days. 0.01 g of each crude extract was stored as a stock solution for bioactivity assays. The crude extracts were stored at -20°C for further studies.

### 5.2. Cell Culture

The cells of the human hepatocellular carcinoma cell line (HepG2), colorectal adenocarcinoma cell line (HCT116), and breast adenocarcinoma cell line (MCF-7) were obtained from the American Type Culture Collection (ATCC). Cells were maintained in RPMI-1640 supplemented with 100 *μ*g/ml penicillin and heat-inactivated fetal bovine serum (10% *v*/*v*) in a humidified 5% (*v*/*v*) CO_2_ atmosphere at 37°C [40].

### 5.3. Cytotoxicity Assessment

The cytotoxicity of different compounds was tested against human tumor cells using the Sulphorhodamine B assay (SRB). Healthy growing cells were cultured in a 96-well tissue culture plate (3000 cells/well) for 24 hours before treatment with the tested extracts to allow attachment of the cells to the plate. Cells were exposed to five different concentrations of each extract (0.01, 0.1, 1, 10, and 100 *μ*g/ml), and untreated cells were included as a control. Triplicate wells were incubated with different concentrations for 72 hours and subsequently fixed with TCA (10% *w*/*v*) for one hour at 4°C. After several washings, cells were stained by 0.4% (*w*/*v*) SRB solution for 10 min in a dark place. Excess stain was washed with 1% (*v*/*v*) glacial acetic acid. After drying overnight, the SRB-stained cells were dissolved in tris–HCl and the color intensity was measured in a microplate reader at 540 nm. The linear relation between the viability percentage of each tumor cell line and extract concentration was analyzed to get the IC_50_ (dose of the drug which reduces survival to 50%) using SigmaPlot 12.0 software [41].

### 5.4. Cell Cycle Distribution Using DNA Flow Cytometry

The cells were treated with the extract IC_50_ of test compounds for 48 hours and collected by trypsinization, washed with ice-cold phosphate buffer saline (PBS), and resuspended in 0.5 ml of PBS. 10 ml of 70% ice-cold ethanol was added gently while vortexing. Cells were kept at 4°C for one hour and stored at -20°C until analysis. Upon analysis, fixed cells were washed and resuspended in 1 ml of PBS containing 50 mg/ml RNase A and 10 mg/ml propidium iodide (PI). After 20 min incubation at 37°C, cells were analyzed for DNA contents by FACSVantage™ (Becton Dickinson Immunocytometry Systems, San Jose, CA). For each sample, 10,000 events were acquired. Cell cycle distribution was calculated using CELLQuest software (Becton Dickinson Immunocytometry Systems, San Jose, CA) [42].

### 5.5. Apoptosis Assessment with Acridine Orange/Ethidium Bromide Staining

The cells were seeded in 6-well tissue culture plates and incubated for 48 hours with the IC_50_ of the compound. Cells were harvested using trypsinization and washed twice with PBS. Ten microliters of the cells was transferred to a glass slide and mixed with 2 *μ*l of AO/EB mixture (100 *μ*g/ml of AO and 100 *μ*g/ml of EB). The apoptotic, necrotic, and live cells were examined immediately under a fluorescence microscope [43].

### 5.6. Western Immunoblotting

The western blotting procedure was performed as described [44]. In brief, cancer cells were treated with respective concentrations (IC_50_) of hexane extract for 48 hours and lysed with cell lysis buffer. The total protein concentration was estimated by Coomassie plus protein assay kit (Pierce; Rockford, IL, USA), and 20–40 *μ*g protein from the cell lysate was fractionated with SDS-PAGE followed by transfer to a nitrocellulose membrane. After probing with the respective primary antibodies, HRP-secondary antibodies were added, and the blots were developed using an enhanced chemiluminescence (ECL) detection kit. The membranes were stripped off the antibodies and incubated with the *β*-actin antibody at 1 : 5000 dilutions and redeveloped to detect the *β*-actin band. Bands specific to Bcl-2, Bax, and caspase-3 were quantified using ImageJ (Ver. 1.46, NIH) and normalized to *β*-actin band intensity. All the experiments were performed in triplicates. For statistical analysis, two-tailed Student's *t*-test of GraphPad Prism 6.0 (La Jolla, USA) was used, and the *P* value of ≤0.05 was considered statistically significant. Caspase-3, Bax, Bcl-2, and *β*-actin antibodies were purchased from Santa Cruz Biotechnology, Santa Cruz, CA, USA.

## Figures and Tables

**Table 1:** The IC_50_ (*μ*g/ml) of different extracts of *Saussurea costus* against different tumor cell lines. Results were expressed as mean ± SD for three different independent replicates.

Extract	MCF-7	HepG2	HCT116
Methanol	1.3 ± 0.2	2.5 ± 0.6	1.7 ± 0.3
Hexane	0.54 ± 0.04	0.5 ± 0.09	0.99 ± 0.09
Chloroform	1.8 ± 0.3	0.8 ± 0.01	2.1 ± 0.18
Ethyl acetate	0.67 ± 0.05	1.2 ± 0.2	0.4 ± 0.05
Butanol	25.5 ± 2.8	33.2 ± 2.5	24.9 ± 3.07
Water	250.31 ± 10.9	380.4 ± 163.7	204.3 ± 65.12
Doxorubicin	0.45 ± 0.0516	0.6 ± 0.022	0.42 ± 0.103

**Table 2:** Cell cycle phase distribution (%) of the tumor cell lines after their treatment with different solvent extracts of *Saussurea costus*. Results were expressed as mean ± SD for three independent replicates.

Tumor cell	Extracts	Cell phases		
		G_1_	S	G_2_/M
MCF-7	Cell control	50.32 ± 0.98	23.21 ± 0.6	26.47 ± 0.9
	Methanol	63.42 ± 1.5	5.87 ± 0.5	30.71 ± 1.01
	Hexane	68.92 ± 0.9	13.45 ± 1.1	17.63 ± 0.52
	Chloroform	64.25 ± 0.5	11.43 ± 0.7	24.32 ± 0.6
	Ethyl acetate	67.82 ± 0.7	12.65 ± 1.1	19.53 ± 0.8
	Butanol	54.71 ± 1.1	12.08 ± 0.5	33.21 ± 0.7

HepG2	Cell control	56.44 ± 1.1	24.58 ± 1.1	18.98 ± 2.1
	Methanol	66.87 ± 1.5	13.76 ± 1.16	19.37 ± 1.1
	Hexane	69.11 ± 2.2	12.12 ± 1	18.77 ± 1.3
	Chloroform	58.25 ± 0.4	18.31 ± 1.4	23.44 ± 1
	Ethyl acetate	56.74 ± 0.8	20.9 ± 1	22.36 ± 1.6
	Butanol	61.39 ± 1.7	16.3 ± 1.7	22.31 ± 0.9

HCT116	Cell control	48.54 ± 2.3	23.13 ± 1.4	28.33 ± 1.1
	Methanol	59.35 ± 1.3	6.91 ± 1	33.74 ± 1.7
	Hexane	68.2 ± 2.7	16.13 ± 1.3	15.66 ± 1.6
	Chloroform	56.8 ± 1.3	11.1 ± 1.4	32.1 ± 0.8
	Ethyl acetate	66.87 ± 1.8	20.48 ± 0.8	12.65 ± 1.5
	Butanol	51.87 ± 2.5	16.61 ± 1.7	31.52 ± 1.4

## Data Availability

All data generated or analyzed during this study are included in this article.
